# Green Supply Chain Management With Sustainable Economic Growth by CS-ARDL Technique: Perspective to Blockchain Technology

**DOI:** 10.3389/fpubh.2021.818614

**Published:** 2022-01-21

**Authors:** Zhenjing Gu, Haider Ali Malik, Supat Chupradit, Gadah Albasher, Vitality Borisov, Natasha Murtaza

**Affiliations:** ^1^Institute of Cultural Industries, Shenzhen University, Shenzhen, China; ^2^Institute for Culture Industries, Shenzhen University, Shenzhen, China; ^3^FAST School of Management, Islamabad, Pakistan; ^4^Department of Occupational Therapy, Faculty of Medical Sciences, Chiang Mai University, Chiang Mai, Thailand; ^5^Department of Zoology, College of Science, King Saud University, Riyadh, Saudi Arabia; ^6^I.M. Sechenov First Moscow State Medical University, Moscow, Russia; ^7^Faculty of Social Sciences, Institute of Agricultural and Resource Economics, University of Agriculture, Faisalabad, Pakistan

**Keywords:** green environment, green logistics performance, CS-ARDL, carbon emissions, sustainable economic growth, STIRPAT, renewable energy resources

## Abstract

Logistics plays a major part in any country's or region's economic success. Logistics performance depends upon the trade between other countries and urbanization. Urbanization has major role in logistics performance. However, being a significant energy user, logistics has negative consequences. As the logistics performance increases, carbon emissions increase as well because of more transportation and urbanization. Logistics performance has positive effects related to trade openness which reduces carbon emissions. As a result, it is necessary to understand function of logistics from both economic and environmental standpoint. Logistics performance is affected by urbanization of any region. The dataset for this research is made up of 10 Asian nations with 550 observations from 2010 to 2018 and is based on the theoretical underpinnings of impact of population affluence and technology (IPAT) and stochastic impacts by regression on population affluence and technology (STIRPAT). After applying various tests like cointegration analysis, unit root test, cross-sectional dependence now long & short-term relation of variables is studied by Cross-sectionally augmented autoregressive distributed lag (CS-ARDL). As indicated by the discoveries, the logistic performance index (LPI) is basically effective on economic growth and carbon emissions, particularly when related to IPAT and STIRPAT. The findings are reviewed, and policy implications are offered, which say that current logistical infrastructure should be transformed to more environmentally friendly operations. Finally, the limits are acknowledged, as well as future research possibilities that should be pursued.

## Introduction

Logistics is management of acquisition, storage of materials, transportation of components and finished products inventories (as well as associated information flow) across companies and marketing platforms (1). Also, logistical expansion may help to boost economic growth, but it can also have a detrimental influence on environment and green energy. Worldwide acquisition requires a huge interest in transportation and an extended lead time, the two of which contrarily affect environment sustainability (2). According to the United Nations (2014), transportation is responsible for about 25% of worldwide carbon emissions, which is expected to rise to 55% by 2040 unless environmental safety measures are taken. Furthermore, since petroleum and fossil fuels account for 96 percent of total energy demand, the transportation sector is heavily reliant on them. Excessive energy use increases energy demand while also increasing greenhouse gas (GHG) emissions. As a result, businesses and decision-makers are more concerned about environmental preservation and long-term growth and notion of green logistics (GL) green environment emerges (3).

The supply chain's logistics division is crucial. As green supply chain management (GSCM) expanded internationally, a growing number of scientists began looking at green logistics. eco-logistics includes economic, sustainable environmental and social aspects (4). It focuses around finding a way to lessen negative natural outcomes and offers practices that guide in the development of society and renewable energy resources.

Logistics performance may affect an organization's or country's productivity and efficiency. Furthermore, a country's logistics play a significant part in evaluating a country's total supply chain quality and performance (5). The integration of multiple processes in logistics management improves the total transition of a product from raw material to completed products simpler, smoother, and more punctual. It primarily entails the coordination of transportation, inventories, warehousing, and information exchange amongst supply chain participants (5). A country's logistics operations will profit in a variety of ways if they are performed effectively. Solid logistics setup, for example, will enable you to lower your stock levels and to save funds on inventory investments. This will assist to speed the delivery of items on time, reducing lead times and lowering warehouse carrying and handling expenses by maintaining the optimal amount of inventory. Furthermore, speedy clearance of the cargo from the port will save the importer money on storage and demurrages, therefore an organization at micro level & nation at macro level may save money on many expenditures by having an effective logistics infrastructure and operations (6).

Furthermore, the transportation and logistics industry contributes greatly to global commerce by decentralizing manufacturing, which may be located in a variety of places and then transported to the point of need, thereby supporting globalization by boosting worldwide trade (7). By directing governments' attention to development of logistical infrastructure, relevant stakeholders can potentially save on various costs associated with imports and exports through process integration, cross-country sharing of benchmarks and best practices and real-time open communication and integration, thus allowing and establishing global trade (8).

According to the aforementioned facts, logistics performance is regarded as a key predictor of the country's economic development, and so has attracted the attention of several scholars and academicians (9). Furthermore, many nations' growth percentages reflect the role of logistics in economic development. For example, logistics contributes 7 and 8% of yearly economic growth in advanced nations like Germany and the United States, respectively, demonstrating the importance of logistics in leveling country's economic development (10).

However, there are certain disadvantages to the logistics industry. While the industry employs around 5% of the workforce, it produces carbon & greenhouse gases into environment (5, 6). Despite the fact that environmental scientists have warned world about changes in earth's environmental conditions, such as unusually high temperatures, more frequent rainfalls, thunderstorms and drought. Despite severe laws enforced by government bodies, environmental cooperation among essential stakeholders is critical for sustainable development, particularly in emerging countries (11).

The World Bank, as one of the most significant and biggest financing, supporting, and financially aiding bodies to developing nations, releases the Logistics Performance Index (LPI) for 150 countries every 2 years (12). Regardless of the way that coordination's execution is viewed as a significant supporter of a country's monetary achievement, the connection among coordination's execution and natural corruption is far from being obviously true. As a result, as the country's most noteworthy energy shopper, it's basic to investigate the connection between coordination's execution and both financial development and natural insurance.

In this paper, we provide effects of logistics performance index (LPI) on the environmental health and economic growth by using modern technique CS-ARDL with various other tests. Our aim of study is to verify that logistics performance affects the green environmental health and economic growth and if has negative effects on sustainable economy or environment how to minimize its effects.

The next section provides literature review of logistics in chosen nations; section Methodology provides methodology; section Results and Discussion provides results and discussions; sections Conclusions and Recommendations and Limitations of the Study provide conclusions and limitations of study, respectively.

## Literature Review

Research in GL operations is based on a variety of different topics. There are three theories that involve reverse logistics, emissions assessment, and the “greening” of supply chains and logistical operations (13). Strategic and operational aspects of green logistics (such as the selection and assessment of a sustainable logistics provider) were highlighted in another research (14). An important area of research in GL operations is the trade-off between economic gain and environmental impact. When it comes to implementing eco-friendly sustainable logistics operations, four obstacles were found: consumer priorities, management complexity, network imbalance and uncertainty over new technical or legal developments as the four main hurdles (15). It has been shown that GL innovation may lower the environmental effect of logistics activities and boost company competitiveness and sustainability (16). Upstream supply chain measures can enforce environmental friendliness on logistics businesses, a downstream supply chain participant (17). Similarly, to be clear, the research cited above focused on ways to reduce environmental deterioration without jeopardizing business interests (18, 19). However, urbanization also effects logistics. Urbanization leads to logistics as the rural areas are developed, logistics performance increases automatically. Also, logistics performance leads to urbanization as there is development of transportation, warehouses and freight forwarders. Both of the factors urbanization and logistics are main features of economic growth and development (20). Relation of logistics to economic growth and environmental factors is discussed below.

### Logistics and Sustainable Economy

In literature, the term transportation and logistics (albeit transportation is component of logistics) is used, but transportation is described as a component that was identified early on as essential component of logistics (21). Logistics has major role in a country's economic development, empirical research on this link are few (5). However, there are four ways in which logistics may assist a country in accelerating its economic development. To begin with, modern logistics infrastructure assists a nation in focusing commercial activities, increasing the economy's total output (22). Second, excellent logistics infrastructures aid in the attraction of foreign direct investment which in turn aids in country's economic development (23). Third, good logistics aids a business in reducing logistical waste and expenses, making them more competitive and boosting the market offers in worldwide market. It aids in better communication between parties, which is a crucial component of logistics (24, 25). Fourth, to maintain updated logistics infrastructure, investments in technology, machinery, and equipment will be made, resulting in the logistics sector indirectly supporting a country's economic development (26).

The LPI proposal has been seen as a step forward in the study of logistics at the macro-level (6, 12). The link between the LPI and other economic indicators has been investigated in a number of researches. For example, a researcher investigated the levels of international commerce while considering customs operations, logistics expenditures, and the soundness of logistical infrastructure LPI, which also emphasizes the initiatives performed by the relevant authorities around the globe. Researchers looked into the association between GDP and global competitiveness index with LPI, whereas relationship with economic growth using data from 32 OECD nations from 1994 to 2011 (27). In 2021, researcher analyzed relation of green supply chain management and governance mechanism. Their study showed that how choice of governance mechanism can affect the companies green supply chain management performance and competition. It showed that formal governance can affect process management (16). In 2021, research was conducted on south Asian countries using panel data estimation. Results revealed that financial development and income inequality have positive effect on economic growth while energy usage has opposite effect (28).

### Logistics and Environment

Logistical services, as previously mentioned, are important users of energy and hence contribute to environmental degradation through the emission of greenhouse gases and pollution. Logistics and operations must be managed by legislation if economies are to be sustainable, and thus compromises the sustainability of the environment (29). Because they are viewed as low cost-bearing investment with minimal economic returns, green practices tend to counter environmental deterioration to a greater extent than those implemented by the United States and the United Kingdom, although these are frequently ignored by countries (11). According to researchers in 2017 who studied trends in 27 European countries, serious measures must be taken by the relevant authorities to control logistics' negative environmental impacts, while green practices must be applied among logisticians for long-term sustainability. Researchers confirmed the link between logistics and carbon emissions in Balkan nations (30). There are number of additional options that might be beneficial in the fight against carbon emissions. Even though it's a cost-effective investment, sustainable logistics also helps companies make more money, while it also improves the company's reputation in the market on all three fronts (ethically, morally, and financially) (31). The authors further argued that for an economy to be sustainable, government institutions must have non-flexible policies in place to reduce greenhouse gas emissions dramatically. This is because environmental degradation is accelerated when foreign direct investments and inflows are made (32). Since the number of vehicles on the road increases on a regular basis, pollution levels will rise and the environment will worsen (33).

In 2018, Generalized Methods of Moments technique was used to analyze green logistics and environmental sustainability. Data was collected from 43 nations and findings revealed that non-renewable energy and fossil fuels used in GL operations have harmful effects on environmental sustainability and economic growth (34). In 2021, researchers in China analyzed 501 samples from enterprises of green supply chain management on clean technology innovation. Small industries have more motivation to adopt green supply chain management than heavy or large industry (35). In 2021, due COVID pandemic study was conducted to analyze the logistics operations linkage with environmental impacts. Panel data was collected from 25 countries and it revealed that LPI has negative effects on economic growth. LPI has positive effects on carbon emissions but green supply chain management is far away from decarbonization (36). Also, in 2021 research was conducted on E7 countries regarding environmental taxes and carbon neutrality. Panel data econometric tools were used to conduct the experiments. Results revealed that environmental taxes and eco-innovation play significant role in carbon emission mitigation (37). In 2021, researchers analyzed the greenhouse gas emissions regarding agricultural and food system using panel regression method on BIMSTEC region. Results showed that renewable energy can help mitigate the pesticides effects on greenhouse gasses (38).

Although the above discussion of logistics' role in sustainable development focused on maintaining right balance between economic growth and environmental degradation. From existing literature, it is clear that logistics not only facilitates economic activity, but also has negative effects on the environment, necessitating further study. With that in mind, it is clear that addressing the issue raised in this study in relation to major Asian countries, whose operationalization will be elaborated on in later parts, makes sense.

## Methodology

This part goes through the data, how to measure it, how to specify the model, and how to make the study practical. Here top 10 Asian countries (China, Hong Kong, Japan, India, Pakistan, Bangladesh, Indonesia, Malaysia, Singapore and South Korea) are selected according to their high population and trading values. Here CE represents carbon emissions, EG represents economic growth, LAB represents labor (working manpower), CPT represents capital, URB represents urbanization and PCI represents per capita income. Because all variables are measured in various units, it's necessary to convert them all to the same unit in order to improve the result and interpretations. All the data is collected from World development indicators (WDI), as given in Table 1.

**Table 1:** Source of all variables.

**Variable**	**Unit**	**Source**
Carbon emissions (CE)	Discharged carbon in K tons	WDI
Economic growth (EG)	Market priced gross domestic product with base of 2015 USD	WDI
Labor (LAB)	Working manpower of population having age of more than 15 years	WDI
Capital (CPT)	Gross formation of fixed capital with base of 2015 USD	WDI
Per capita income (PCI)	Income of household	WDI
Urbanization (URB)	Total number of people from population living in urban cities	WDI
Population (PPL)	Entire people living in the nation	WDI
Logistics performance index (LPI)	Overall logistics of nation	WDI

Firstly, we are going to specify a model for economic growth and carbon emissions which will be related to logistics performance. After that we will apply unit root test to verify stationarity in the data set. After that cointegration test will be performed along with panel cointegration and slope heterogeneity test to verify the data set of countries for EG and CE variables. At last we will perform CS-ARDL log-run and short-run test to verify that what effects logistics have on economic growth and carbon emissions.

According to the researcher, natural logarithm was used to reduce difficulties with distributions within the data set. Furthermore, the derived coefficients from natural log, indicate elasticity that improves quality of interpretations (39).

### Model Specifications

To achieve the study's goal of exploring role of logistics in environmental and economic aspects, modeling is build based on 2 theories that give theoretical foundations: the neoclassical growth model & IPAT model for determining economic and environmental outcomes (40). Furthermore, urbanization was utilized as a control variable since the degree and quality of logistical infrastructure affects the probability of urban intensity. Nonetheless, the models are discussed in more detail below:

The following model is presented for analyzing the influence of LPI and URB on EG:


(1) $$\begin{array}{l} EG_{it}=f\left(LAB_{it},LPI_{it},CPT_{it},URB_{it},\;v_{i}\right) \end{array}$$

In Equation 1, EG represents economic growth, CPT represents capital, LAB represents labor, URB represents urbanization, LPI represents logistics performance index, and vi represents the nation's fixed impact, whilst (t) and (i) indicate time period and country, respectively. Furthermore, IPAT model was used to examine the impacts on environmental elements, as it has been in previous studies (41, 42). The following is based on this model's underlying link between money, technology, population, and environment:


(2) $$\begin{array}{l} I=A\;\times T\;\times P \end{array}$$

In Equation 2, I represent pollution, which is calculated from consumption (A), technical efficiency (T) and population (P), which is defined as per unit of pollution. This is an early model that was developed into a stochastic version Dietz and Rosa known as STIRPAT (Stochastic Impacts via Regression on Population, Affluence, and Technology) (43). As a result, the following equation is suggested based on theoretical underpinning of STIRPAT approach:


(3) $$\begin{array}{l} CE_{it}=f\left(PCI_{it},LPI_{it},PPL_{it},URB_{it},\;v_{i}\right)\; \end{array}$$

Equation 3 shows how to calculate CE by managing the impact of logistics performance, urbanization, population and per capita income with other effective factors on country or global level. LPI represents logistics performance index, PCI represents per capita income, PPL represents population of each nation, URB represents urbanization of each nation and v represents other factors while (t) and (i) represent time period and nation.

### Unit Root Test

The current study's research began with a determination of unit's cross-sectional dependency (CD). For determining specific unit roots tests from various possibilities to treat cross-sectional dependency, evaluating CD before analyzing unit root is advantageous. When a research is conducted on a data set of sections with comparable areas or locations, some elements, such as economic crises, inter region regulations and oil prices have an overall influence on the region. Consequently, if the CD is not appropriately handled, inaccuracies in the estimates may arise, leading to incorrect conclusions and interpretations (44, 45). Several studies have investigated stationarity (46–48). There are a number of tests available for determining unit root, but each has its own set of advantages and disadvantages. Three generations have been established. This classification is also based on the ability to deal with challenges in various scenarios. For example, in homogeneous panel estimates, non-stationary may be tested using the methods suggested (48).

Test categorizes in second generation Moon and Perron, on the other hand show that even in case of heterogeneous panel estimates, CD may be addressed (49). Although both 1st and 2nd generation tests are incapable of integrating structural breakdowns, they lose their prediction power as a result. Because of its capacity to include structural breakdowns while also addressing heterogeneity and CD, third generation tests in order to solve non-stationarity problem and cross-sectional dependence (49).

### Cointegration Test

After determining stationarity, modified Swamy's test is used to determine the slope's heterogeneity (50). The null hypothesis represents the existence of homogeneous slope, while alternative hypothesis represents presence of a heterogeneous slope. Because standard tests fail to provide credible estimates when CD is present, the current research uses tests by Banerjee and Carrion-i-Silvestre (57) and Westerlund and Edgerton (51). Tests developed are capable of addressing issues such as slope heterogeneity, lack of stationarity, most crucially, including structural discontinuities without affecting slope homogeneity and refusing null hypothesis (51). Swamy's test modified version for slope hetrogenity test is as followed.


(4) $$\begin{array}{ll} Ŝ & =\;\sum_{i=1}^{N}(\hat{\beta }-\hat{\beta_{WFE}}{)}^{\prime }\;\frac{{X^{{\;}^{\prime }}}_{i}M_{t}X_{i}}{{\hat{\alpha }}_{i}^{2}}\;\left(\hat{\beta }-\hat{\beta_{WFE}}\right) \end{array}$$

(5) $$\begin{array}{ll} {\hat{\alpha }}_{i}^{2} & =\;\frac{{\left(y_{i}-X_{i}{\hat{\beta }}_{i}\right)}^{{\;}^{\prime }}M_{t}\;\left(y_{i}-X_{i}{\hat{\beta }}_{i}\right)}{\left(T-k-1\right)} \end{array}$$

(6) $$\begin{array}{ll} {\hat{\beta }}_{WFE} & =(\sum_{i=1}^{N}\frac{{X^{{\;}^{\prime }}}_{i}M_{t}X_{i}}{{\hat{\alpha }}_{i}^{2}}{)}^{-1}\;\sum_{i=1}^{N}\frac{{X^{{\;}^{\prime }}}_{i}M_{t}X_{i}}{{\hat{\alpha }}_{i}^{2}} \end{array}$$

Here, N is fixed and T is for infinity. K(N-1) is degree of freedom.

### CS-ARDL Test

When investigating any phenomena using a dataset including portions from same region, the CD remains a difficulty because to the need for consistency while dealing with regional concerns such as oil price changes, financial disturbances, and so on. Because of significant correlation in unobserved variables, CS-ARDL is favored for addressing CD and slop variability between sections, which may result in worse findings. The CS-ARDL calculations are based on dynamic common correlated effects and begin as follows:


(7) $$\begin{array}{l} W_{i,t}=\;\sum_{I=0}^{p_{w}}\gamma \;I,i\;W_{i,t-1}+\;\sum_{I=0}^{p_{z}}\beta \;I,i\;Z_{i,t-1}+\;\varepsilon_{i,t} \end{array}$$

When CD is present, Equation (7) reflects Autoregressive Distributed Lags (ARDL) model, however when Equation (8) is used, it is possible to get incorrect results. Equation (8) is modification of Equation (4), in which the cross-section averages of each independent variable is employed to help overcome the unfitting inference while taking into account the existence of the needed effect due to the CD (52).


(8) $$\begin{array}{l} W_{i,t}=\;\sum_{I=0}^{p_{w}}\gamma \;I,i\;W_{i,t-1}+\;\sum_{I=0}^{p_{z}}\beta \;I,i\;Z_{i,t-1}\\ \;+\sum_{I=0}^{p_{x}}\alpha^{\prime }\;I,i\;{\overset{-}{X}}_{i,t-1}\;+\;\varepsilon_{i,t} \end{array}$$

*X*_*t*1_ = *W*_*i*,*t*−*1*_, *Z*_*i,t*−1_ represents average of both independent and dependent variables in Equation (5), whilst *p*_*w*_, *p*_*z*_, *p*_*x*_ represents the delays for all variables. Furthermore, in the current research, *Z*_*i,t*_ represents all independent factors, including URB & LPI, while *W*_*i,t*_ represents all dependent variables, including EG and CE. For this reason, the average over sections is denoted by X, which eliminates the possibility of spill-over dependency (53). Long-run CS-ARDL coefficient estimations are based on short-run CS-ARDL coefficient estimates, which should be noted. The following table includes the long-run coefficients and the mean group estimator:


(9) $$\begin{array}{l} \overset{-}{\pi }\;CS-ARDL,i=\;\frac{\sum_{I=0}^{pz}\hat{\beta }I,i^{pw}}{1-\;\sum I=0}\;\hat{\gamma \;I,i} \end{array}$$

And also,


(10) $$\begin{array}{l} \hat{\overset{-}{\pi }}M,G=\;\frac{1}{N}\;\sum_{i=1}^{N}\hat{\pi }\;i \end{array}$$

(11) $$\begin{array}{l} \Delta W_{i,t}=\vartheta_{i}\left[W_{i,t-1}-\pi_{i}Z_{i,t}\right]-\sum_{I=0}^{p_{w-1}}\gamma \;I,i\Delta_{I}W_{i,t-1}\\ \;+\sum_{I=0}^{p_{z}}\beta \;I,i\Delta_{I}Z_{i,t}+\sum_{I=0}^{p_{x}}\alpha^{\prime }I,i{\overset{-}{X}}_{t}+\varepsilon_{i,t} \end{array}$$

Also,


(12) $$\begin{array}{l} \hat{T_{i}}\;=-\left(1-\sum_{I=1}^{pw}\hat{\gamma \;I,i}\right) \end{array}$$

## Results and Discussion

### Results of Cross-Sectional Dependence Analysis

**Figure 1:**
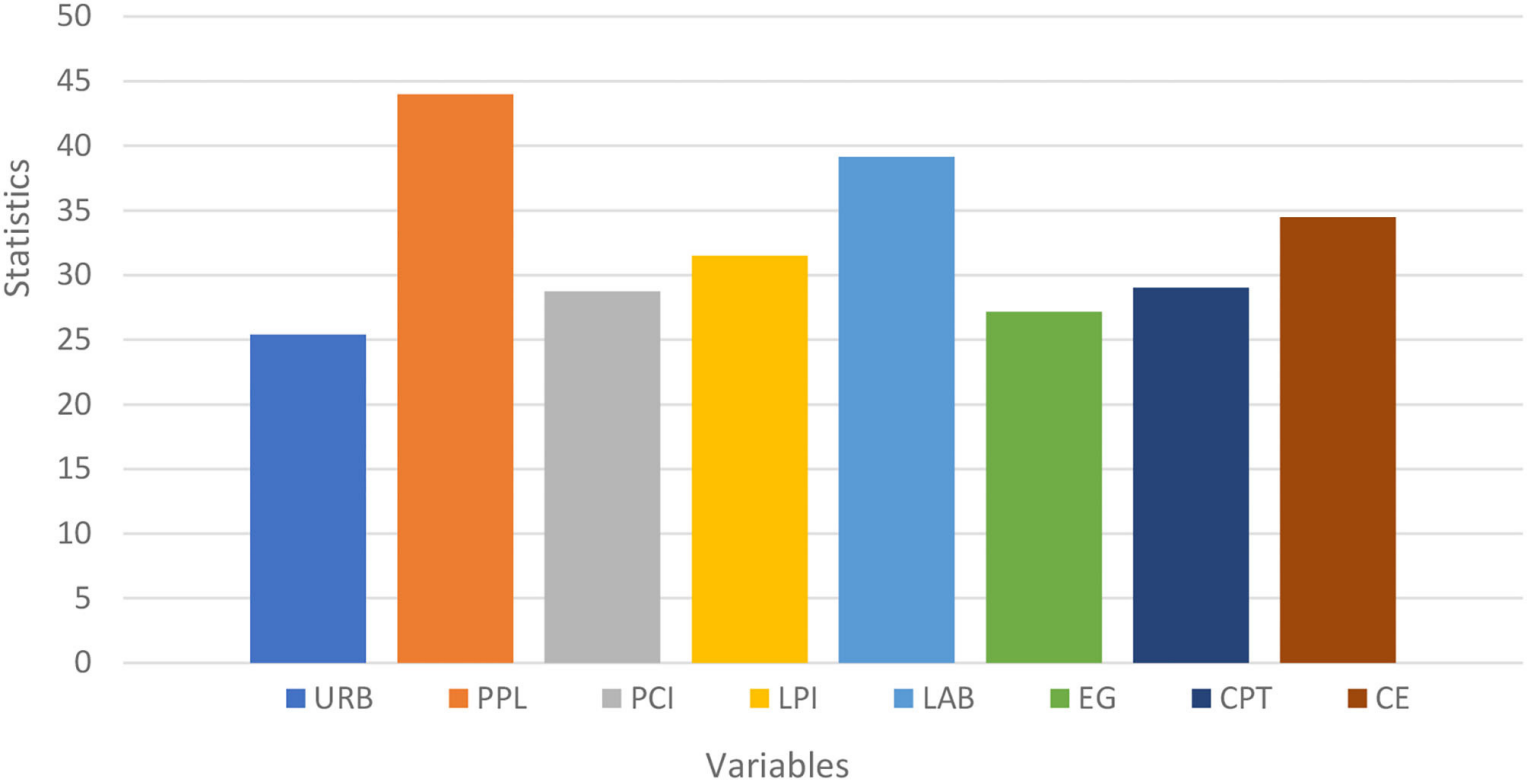
Results of cross -sectional dependence analysis.

The CD test, as stated in the preceding section, is critical for providing impartial findings; if it is not examined, the succeeding tests will provide incorrect stationary and unit root results. All of the study variables concentrated on factors have huge *p*-values at the 1 percent level of importance, dismissing the invalid theory that there is no cross-sectional reliance between them. This shows that there is cross-sectional reliance between them in the informational collection. Table 2 sums up the consequences of the cross-sectional reliance test dependent on Psarian's test (54). Figure 1 shows statics values of each variable from the cross-sectional dependence test.

**Table 2:** Results of cross-sectional dependence analysis.

**Variable**	**Statistics (*p*-values)**
URB	25.386***
PPL	43.986***
PCI	28.749***
LPI	31.489***
LAB	39.147***
EG	27.157***
CPT	29.045***
CE	34.489***

10, 5, and 1% of significance level is shown, respectively, by *, ** and ***.

### Results of Unit Root Test

**Figure 2:**
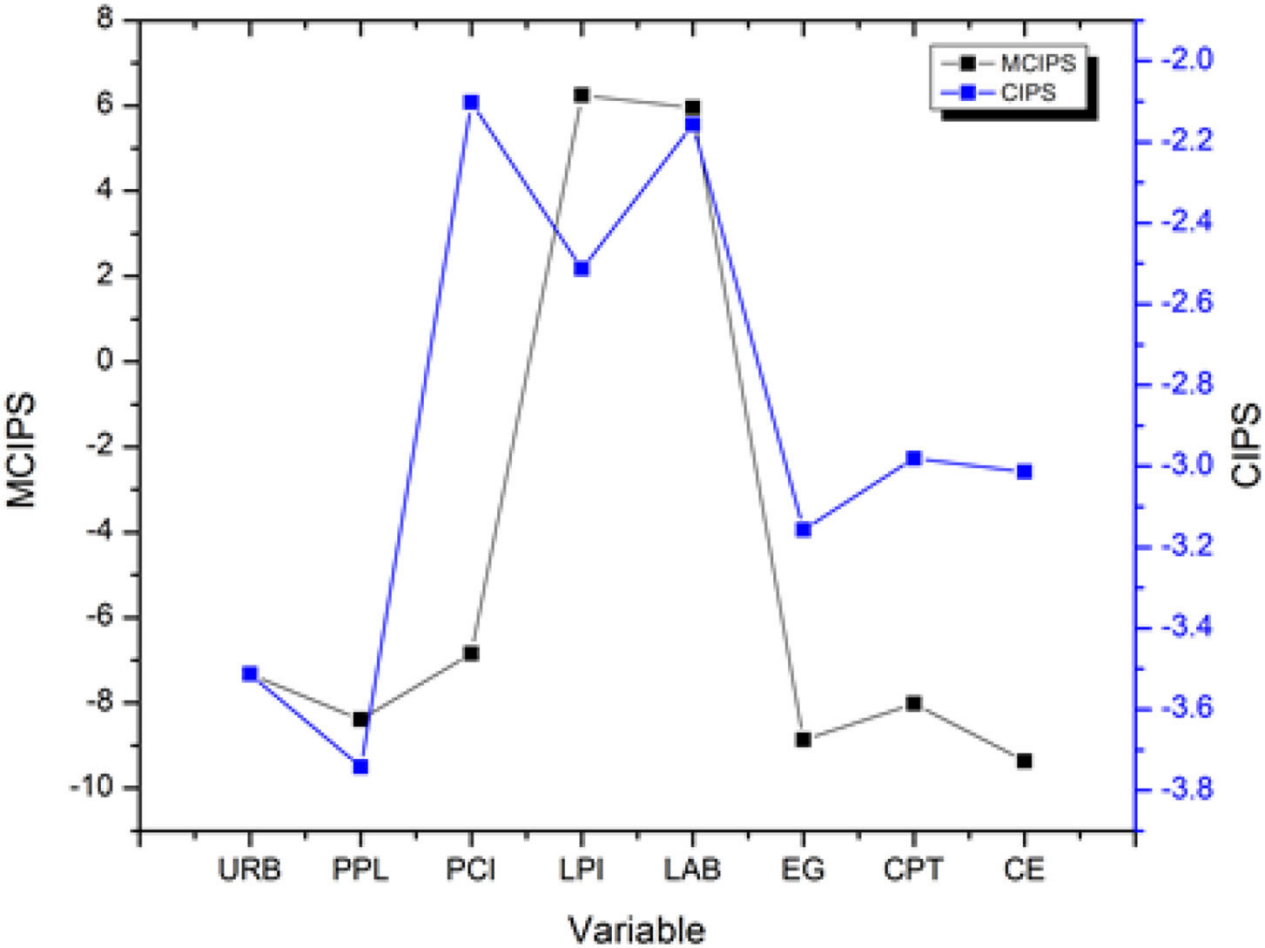
Results of unit root test (Level 0); Pesaran (49).

**Figure 3:**
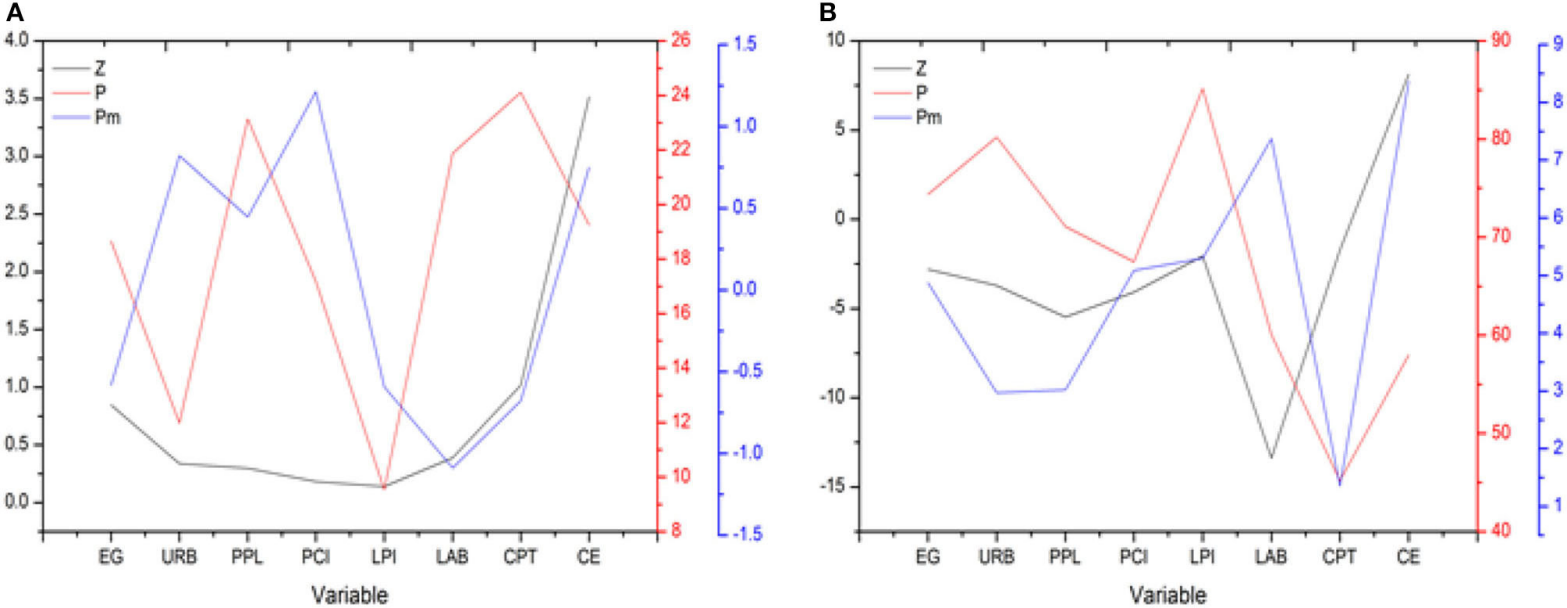
Results of unit root test **(A)** Level (0); **(B)** First difference (1); Bai and Carrio n-i-Silvestre (55).

Findings reveal that null hypothesis is rejected while accepting existence of unit root in presence of cross-sectional dependence, heterogeneous slope, and structural breaks. As a result, every variable investigated was determined to be stationary and/or integrated. Table 3 shows a summary of the findings. Figure 2 shows the results of unit toot test by Pesaran with CIPS and MCIPS at level (0) (55). Figure 3 shows the results of unit root test by Bai el al. with level (0) and first difference level.

Table 3Result of unit root test.

**Table:** 

**Variable**	**MCIPS (Level 0)**	**CIPS (Level 0)**
**Pesaran** **(****49****)**		
URB	−7.326**	−3.513***
PPL	−8.391**	−3.742^***^
PCI	−6.846*	−2.102**
LPI	6.248*	−2.513**
LAB	5.957*	−2.157**
EG	−8.87**	−3.157***
CPT	−8.02**	−2.981***
CE	−9.36**	−3.014***

**Table:** 

	**Level (0)**			**First level**		
**Variable**	***Z***	***P***	***P*_m_**	***Z***	***P***	***P*_m_**
**Bai and Carrio n-i-Silvestre (55)**						
EG	0.846	18.662	−0.583	−2.795***	74.349***	4.879***
URB	0.338	11.978	0.821	−3.715***	80.189***	2.971***
PPL	0.297	23.143	0.446	−5.478***	71.088***	3.018***
PCI	0.184	17.179	1.214	−4.089***	67.489***	5.089***
LPI	0.141	9.541	−0.591	−2.049**	85.146***	5.300***
LAB	0.389	21.879	−1.088	−13.384***	60.054***	7.371***
CPT	1.018	24.113	−0.679	−1.708**	45.189*	1.348*
CE	3.516	19.241	0.751	8.136***	58.015***	8.367***

10, 5, and 1% of significance level is shown, respectively, by *, ** and ***. CIPS means chartered institute of procurement and supply. MCIPS means members of chartered institute of procurement and supply.

### Results of Slope Heterogeneity Test

The slope's heterogeneity was then assessed using homogeneity slope, which Pesaran and Yamagata updated and discussed in 2008 (50). It should be emphasized that if the slope's heterogeneity is not examined, the subsequent analysis will provide incorrect results, contaminating the findings. In this test, null hypothesis asserts that slope's coefficients are homogeneous, while alternative hypothesis suggests that slope is heterogeneous. One percent significance threshold revealed the *p*-values to be very significant, disproving the null hypothesis and empirically confirming that slope coefficients can be heterogeneous. To account for slope heterogeneity, it is vital to employ conventional approximation methods; otherwise, the conclusions may be inconsistent. Term “slope heterogeneity” refers to fact that coefficient is not same for each cross section (56). Table 4 shows a summary of the findings.

**Table 4:** Results of slope heterogeneity test.

**Statistics**	***P*-value**
**DV (Dependent variable): EG**	
	19.583*** (0.000)
Adjusted	20.056*** (0.000)
**DV (Dependent variable): CE**	
	21.969*** (0.000)
adjusted	22.952*** (0.000)

10, 5, and 1% of significance level is shown, respectively, by *, ** and ***.

### Results of Panel Cointegration Analysis

This test examines whether or not there is cointegration, with the null hypothesis stating there isn't cointegration when CD is present in data and the alternative hypothesis suggesting otherwise (51). All three phases of the test (mean shift, regime shift, and no break) reject the null hypothesis at a 1 percent level of significance for both of the research's criterion variables, gross domestic product and carbon emissions. This indicates the presence of cointegration. Table 5 shows a summary of the findings.

**Table 5:** Results of panel cointegration analysis.

**Test**	**No break**	**MS (Mean shift)**	**RS (Regime shift)**
**DV (Dependent variable): EG**			
ZϕN	−3.933***	−3.632***	−4.102***
Pvalue	0.000	0.000	0.000
ZτN	−3.876***	−3.502***	−3.665***
*P*-value	0.000	0.000	0.000
**DV (Dependent variable): CE**			
ZϕN	−3.954***	−4.107***	−3.643***
Pvalue	0.000	0.000	0.000
ZτN	−3.781***	−4.253***	−3.127***
*P*-value	0.000	0.000	0.000

10, 5, and 1% of significance level is shown, respectively, by *, ** and ***.

**Figure 4:**
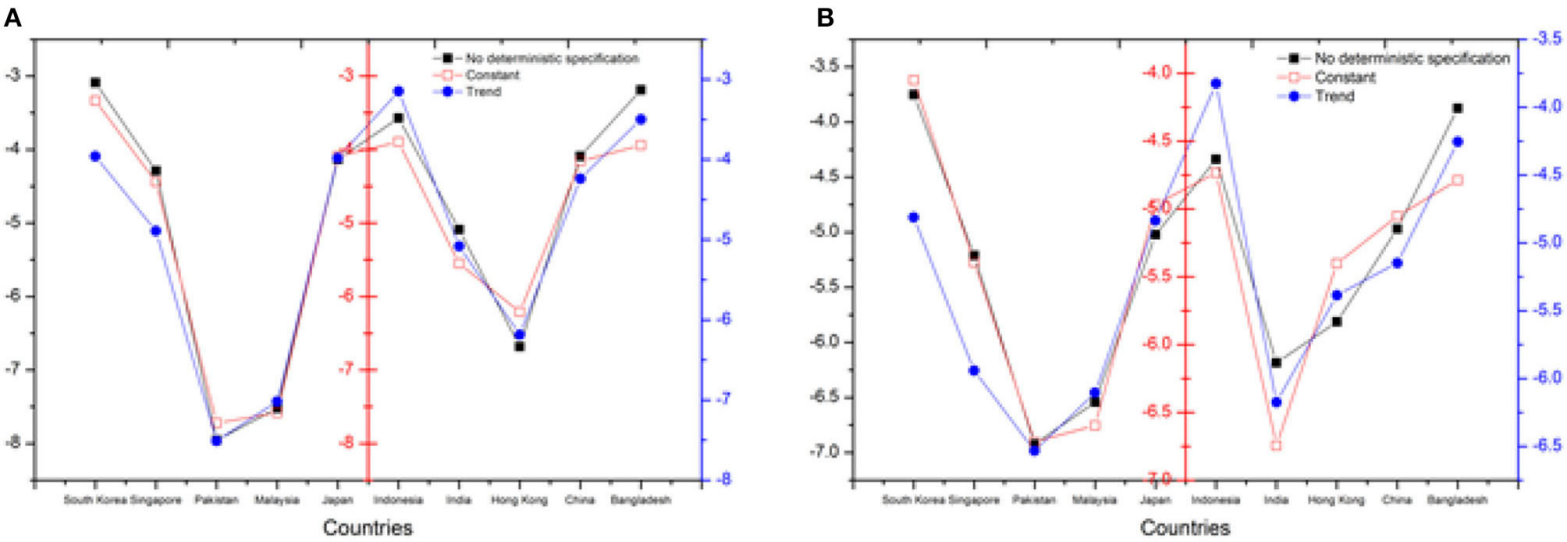
Results of Banerjee and Carrion-i-Silvestre (57) cointegration analysis **(A)** DV: EG, **(B)** DV: CE.

Here, we are going to discuss the cointegration analysis derived by researchers (Banerjee and Carrion-i-Silvestre) in 2017. Cointegration analysis was performed for EC and CE for 10 ASIAN countries. By rejecting null hypothesis for both study criteria variables (EG and CE), the cointegration test shows that there is cointegration (57). The analysis for all of the nations analyzed in this research was determined to be significant at the 1% level of significance, as shown in Table 6 and Figure 4.

**Table 6:** Results of Banerjee and Carrion-i-Silvestre (57) cointegration analysis.

		**DV: EG**			**DV: CE**		
**Sr. No.#**	**Countries**	**No deterministic specification**	**Constant**	**Trend**	**No deterministic specification**	**Constant**	**Trend**
1	South Korea	−3.089**	−3.334**	−3.961**	−3.753**	−4.051**	−4.812**
2	Singapore	−4.289**	−4.441**	−4.891**	−5.211**	−5.395**	−5.942**
3	Pakistan	−7.955**	−7.721**	−7.510**	−6.923**	−6.717**	−6.533**
4	Malaysia	−7.518**	−7.582**	−7.018**	−6.541**	−6.596**	−6.105**
5	Japan	−4.134**	−4.089**	−3.981**	−5.022**	−4.968**	−4.836**
6	Indonesia	−3.571**	−3.897**	−3.149**	−4.338**	−4.734**	−3.826**
7	India	−5.089**	−5.551**	−5.083**	−6.183**	−6.744**	−6.175**
8	Hong Kong	−6.681**	−6.211**	−6.184**	−5.813**	−5.403**	−5.386**
9	China	−4.089**	−4.159**	−4.238**	−4.968**	−5.053**	−5.149**
10	Bangladesh	−3.189**	−3.94**	−3.498**	−3.874**	−4.787**	−4.256**

10, 5, and 1% of significance level is shown, respectively, by *, ** and ***.

### Results of CS-ARDL Long-Run Test

**Figure 5:**
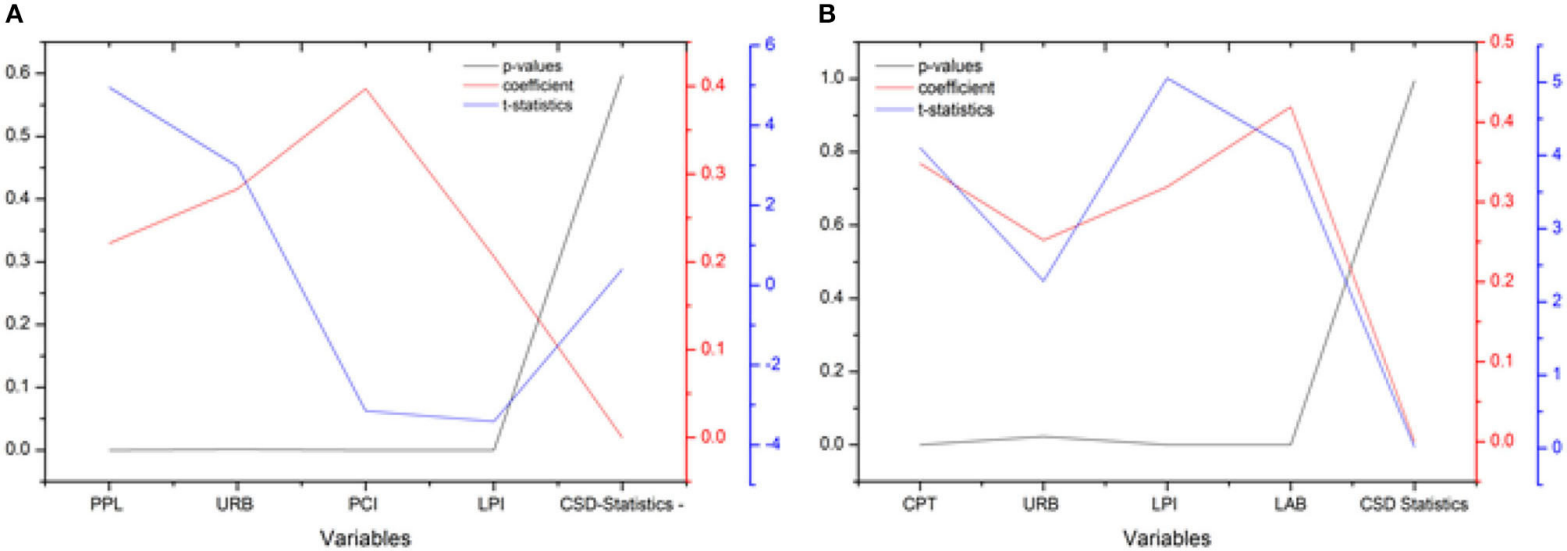
Results of CS-ARDL Long run test **(A)** DV: EG, **(B)** DV: CE.

Once the data shows the existence of cointegration, CS-ARDL may be used to examine the short and long-run relationships of economic growth and carbon emissions, as well as their drivers, using the theoretical channel outlined previously. When looking at long-run associations for economic growth, it was discovered that capital has significant link, which is significant at 1% level of significance and positive with a coefficient value of 0.348, implying that a 1% rise in capital would raise economic growth by 0.348 percent, as given in Figure 5. Second, labor was shown to have a substantial association with economic growth in the long term, with a coefficient value of 0.419, implying that a one percent rise in labor would raise economic growth by 0.419 percent. A substantial link between urbanization and economic growth was found, with a coefficient value of 0.252, meaning that a one-percent increase in urbanization would enhance economic growth by 0.252%. This is noteworthy at the five-per cent level of importance and positive over the long haul. In particular, the coordination's execution list was found to have a solid connection with financial development, with a coefficient worth of 0.319, suggesting that a 1% expansion in the coordination's execution list would raise monetary development by 0.319 per cent. This connection is critical at the 1% degree of importance and positive over the long haul.

When looking at long-run associations for CE, it was discovered that PPL has a significant link that is significant at 1% level of significance and positive with a coefficient value of 0.221, implying that a 1% rise in population would raise carbon emissions by 0.221 percent. Second, per capita index was shown to have substantial association with carbon emissions in the long term, with a coefficient value of 0.397, implying that a one percent rise in per capita index would lower carbon emissions by 0.397 percent. Urbanization was also shown to have a strong link to carbon emissions, with a long-term significance level of 5% and a coefficient value of 0.2833. This means that for every 1 percent increase in urbanization, there will be an equal and opposite increase in carbon emissions of 0.2833 percent.

Most pertinently, the logistics performance index was found to have a strong correlation with carbon emissions, which is huge at a 1% degree of importance and negative over the long haul, with a coefficient worth of 0.206. This implies that a 1% expansion in the coordination's execution file would bring about an increment in fossil fuel byproducts of 0.206. Table 7 shows a summary of the findings.

**Table 7:** Results of CS-ARDL long run test.

	***p*-values**	**Coefficient**	***t*-statistics**
**DV: EG**			
CPT	0.000	0.348	4.105
URB	0.023	0.252*	2.281
LPI	0.000	0.319**	5.056
LAB	0.000	0.419*	4.084
CSD-Statistics	0.995	–	0.019
**DV: CE**			
PPL	0.000	0.221*	4.952
URB	0.001	0.283**	2.973
PCI	0.000	0.397*	−3.148
LPI	0.000	0.206*	−3.411
CSD-Statistics	0.597	–	0.401

10, 5, and 1% of significance level is shown, respectively, by *, ** and ***.

### Results of CS-ARDL Short-Run Test

According to research on short-term economic growth connections (i.e., the relationship between capital and growth), a 1 percent increase in capital raises economic growth by 0.159 percent, giving a coefficient value of 0.159.

Second, labor was shown to have a substantial association with economic growth in the short-run, with coefficient value of 0.196, implying that a one-percent rise in labor would raise economic growth by 0.196 percent. Furthermore, urbanization was shown to have strong association with economic growth in the short term, with a coefficient value of 0.106, implying that 1% rise in urbanization would raise economic growth by 0.106 percent. Most crucially, logistics performance index was shown to have a strong association with economic growth in the short-run, with a coefficient value of 0.205, implying that 1% rise in logistics performance index would raise economic growth by 0.205 percent.

**Figure 6:**
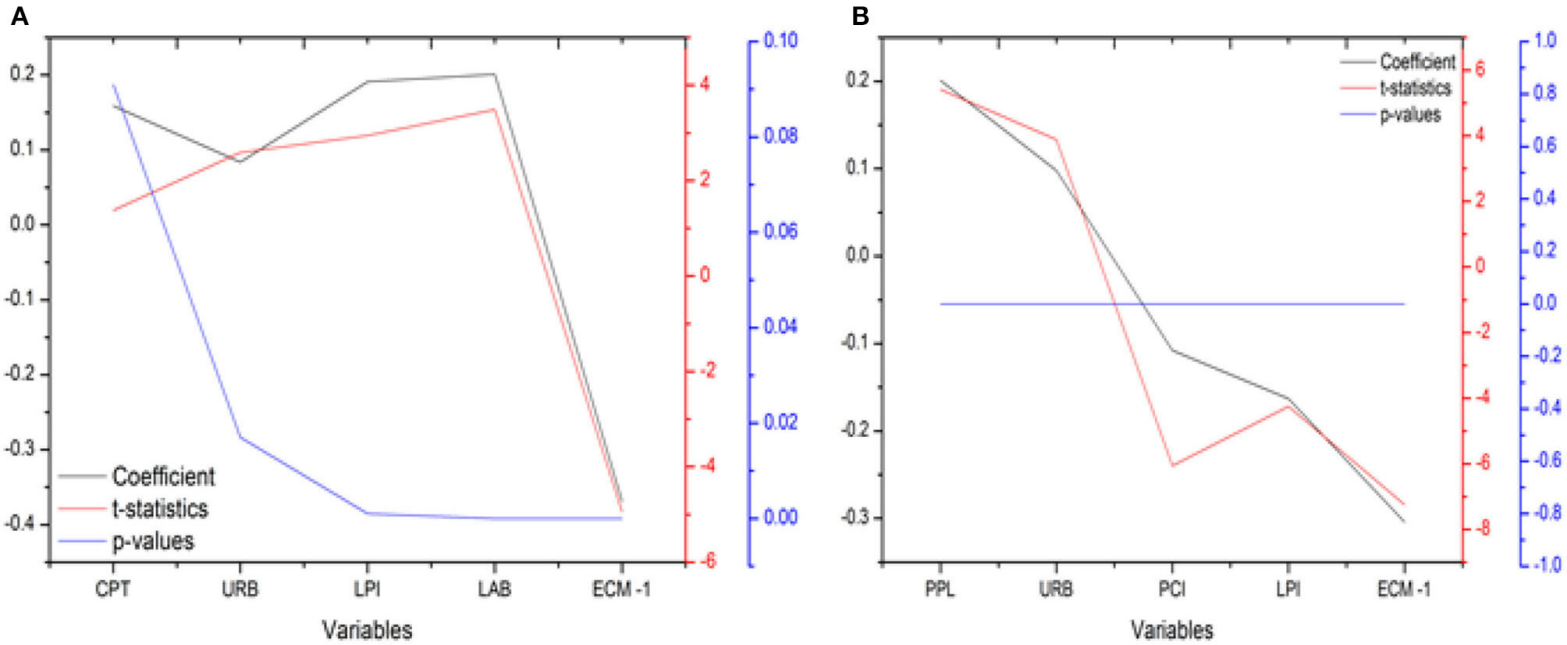
Results of CS-ARDL Short run test **(A)** DV: EG, **(B)** DV: CE.

As Figure 6 elucidates, short-run associations for carbon emissions, it was discovered that population has a significant link that is significant at a 1% level of significance with a coefficient value of 0.201, implying that 1% rise in population would enhance carbon emissions by 0.201 percent. Second, per capita income was shown to have substantial association with carbon emissions in short-run, with a coefficient value of 0.108, implying that 1% rise in per capita index would result in a 0.108 percent drop in carbon emissions. Furthermore, urbanization was shown to have substantial association with carbon emissions in short-run, with coefficient value of 0.098, implying that a 1% rise in urbanization would raise carbon emissions by 0.098 percent. In particular, the logistics performance indicator was shown to have a strong correlation with carbon emissions, with a short-term coefficient value of 0.163 indicating a significance level of 1 percent. This implies that a 1% rise in logistics performance index will raise carbon emissions by 0.163 percent. Tables 7 and 8 represent the summary of these findings.

**Table 8:** Results of CS-ARDL short run test.

**Variables**	**Coefficient**	***t*-statistics**	***p*-values**
**DV: EG**			
CPT	0.159*	1.379	0.091
URB	0.084**	2.587	0.017
LPI	0.191***	2.943	0.001
LAB	0.201***	3.489	0.000
ECM -1	−0.369***	−4.946	0.000
**DV: CE**			
PPL	0.201***	5.421	0.000
URB	0.098***	3.894	0.000
PCI	−0.108***	−6.059	0.000
LPI	−0.163***	−4.249	0.000
ECM -1	−0.304***	−7.246	0.000

10, 5, and 1% of significance level is shown, respectively, by *, ** and ***.

## Conclusions and Recommendations

The research verifies the importance of LPI in complementing EG and combating CE, also the other theoretical IPAT and STIRPAT determinants by basing it on them and using the CS-ARDL that was implemented after numerous preliminary tests had been carried out. To strengthen their logistical infrastructure, governments must adopt cutting-edge technologies like block chain, data analytics, and the psychology of the fourth industrial revolution, based on outcomes and relevance in the Asian area worldwide.

Logistics performance may affect an organization's or country's productivity and efficiency. An organization at micro level and a nation at macro level may decrease costs by reducing expenditures while increasing productivity with an effective logistics infrastructure and operations (6). The transport and logistics industry contribute considerably to global commerce by decentralizing manufacturing, and may take place in a variety of locales, and hence logistics is seen as a key predictor of a country's economic progress. While logistics is critical to country's economic progress, it is also source of carbon emissions, eroding environment's sustainability. Thus, sustainable equilibrium must be established in terms of growth and advancement, but not at expense of environment. The outcomes of study demonstrate critical significance of logistics in ensuring sustainability. These findings are comparable with prior study, in which several researchers agreed on importance of logistics to an economy's financial well-being and also expressed worry about environmental sustainability (5, 58). Nonetheless, by utilizing reasonably sophisticated statistical approach, present study adds to existing literature and verifies findings of earlier studies.

However, since the logistics industry uses a considerable amount of global energy, it has socioeconomic and environmental ramifications. Because of the discussions, the flow research investigated the connection among LPI and EG and CE with regards to 10 Asian countries. Since Asia is the world's biggest and most crowded mainland, and the vast majority of its nations are associated with and have assumed a critical part in worldwide exchange, especially nations like India, turkey and China which are vital participants around here as far as financial action age.

Furthermore, world is now paying attention to developments and changes in Asian trade. Initiative took by China toward the green logistic by offering policy of One Belt, One Road project that ties many regions together by oceans and land for more economic growth and environmental sustainability. As a result, more business may be achieved by removing non-value-added logistical activities and processes, as well as their associated procedures and personnel (59). On the other hand, law enforcement authorities should regulate degree of pollution created by logistics while eliminating pollution via essential innovative technology implementation in order to protect the environment. Specifically, nations must have an environmental orientation to assist them in changing their present infrastructure into one that is more environmentally friendly. Shifting to renewable energy inputs, optimizing current resource usage, and increasing the efficiency of logistical operations are some of the recommendations that will aid nations in preserving the balance of economic development and environmental protection.

Green logistics is rapidly being considered as critical to the creation of sustainable products. Due to the significant degree of interdependence amongst supply chain partners, a business needs communicate information with them in order to manage product development processes properly. Thus, when there is little or no information flow between supply chain participants, green logistics project is almost certain to fail. Typically, environmental effect of goods is considered during the raw material selection process. Managing sources early in design process is critical for developing eco-friendly goods. Indeed, relational supervisory activities have been identified as critical component for product innovation cooperation (16). Companies may engage in global supply chains and collaborate closely with a variety of partners by utilizing collaborative product design. As a result, businesses may gain useful feedback for product design. Additionally, businesses may plan and monitor their environmentally friendly products through collaborative connections. This conclusion corroborates prior research arguing that close relationships between Original equipment manufacturers and vendors help revitalize new product development.

## Limitations of the Study

Based on the limitations of this study, it's been suggested that future research focus on the LPI's green side and the role that individual nations have played in greening logistics. It is also important to look at the non-linear relationship between the variables, which was not examined in this study. For this reason, as well as the fact that logistics were critical in Eastern Europe, these phenomena should be examined across additional panel estimates based on data from diverse locations. It should also be investigated at the individual level by applying various concepts such as the use in decision making of many criteria, mathematical model for structural equation and artificial neural network amongst others.

## Data Availability Statement

The original contributions presented in the study are included in the article/supplementary material, further inquiries can be directed to the corresponding author/s.

## Author Contributions

ZG conceived the main idea and suggested the methodology. HM collected the data for analysis. SC and GA finalized the manuscript. All authors are agreed for publication.

## Conflict of Interest

The authors declare that the research was conducted in the absence of any commercial or financial relationships that could be construed as a potential conflict of interest.

## Publisher's Note

All claims expressed in this article are solely those of the authors and do not necessarily represent those of their affiliated organizations, or those of the publisher, the editors and the reviewers. Any product that may be evaluated in this article, or claim that may be made by its manufacturer, is not guaranteed or endorsed by the publisher.

## References

[1] Christopher M. Logistics and Supply Chain Management. Harlow: Financial Times/Irwin Professional Pub (1992).

[2] Khan SAR Dong QL Yu Z. Research on the measuring performance of green supply chain management: in the perspective of China. Int J Eng Res Afr. (2016) 27:167–78. 10.4028/www.scientific.net/JERA.27.167

[3] Wu H-Y Tsai A Wu H-S. A hybrid multi-criteria decision analysis approach for environmental performance evaluation: an example of the TFT-LCD manufacturers in Taiwan. Environ Eng Manag J. (2019) 18:597–616. 10.30638/eemj.2019.056

[4] Mintcheva V. Indicators for environmental policy integration in the food supply chain (the case of the tomato ketchup supply chain and the integrated product policy). J Clean Prod. (2005) 13:717–31. 10.1016/j.jclepro.2004.01.008

[5] Khan SAR Jian C Zhang Y Golpîra H Kumar A Sharif A. Environmental, social and economic growth indicators spur logistics performance: from the perspective of South Asian Association for Regional Cooperation countries. J Clean Prod. (2019) 214:1011–23. 10.1016/j.jclepro.2018.12.322

[6] Rashidi K Cullinane K. Evaluating the sustainability of national logistics performance using Data Envelopment Analysis. Transport Policy. (2019) 74:35–46. 10.1016/j.tranpol.2018.11.014

[7] Gani A The logistics performance effect in international trade. The Asian Journal of Shipping and Logistics, 2017. 33:279–288. 10.1016/j.ajsl.2017.12.012

[8] Dee P Findlay C Pomfret R. Trade facilitation: what, why, how, where and when? 2006.

[9] Katrakylidis I Madas M. International trade and logistics: an empirical panel investigation of the dynamic linkages between the logistics and trade and their contribution to economic growth. Int Econ Business Administr. (2019) 7:3-21. 10.35808/ijeba/328

[10] Sedgwick SM Mitra S Cooper C. Oil and Gas in California: The Industry and Its Economic Contribution in 2015. Los Angeles, CA: Institute of Applied Economics, Los Angeles County Economic Development Corporation (2017).

[11] Ahmed W Ashraf AS Khan SA Kusi-Sarpong S Arhin FK Kusi-Sarpong H. Analyzing the impact of environmental collaboration among supply chain stakeholders on a firm's sustainable performance. Operat Manag Res. (2020) 13:4–21. 10.1007/s12063-020-00152-1

[12] Arvis J-F Mustra MA Ojala L Shepherd B Saslavsky D. Connecting to Compete 2010: Trade Logistics in the Global Economy–The Logistics Performance Index and Its Indicators. Washington, DC: World Bank (2010). 10.1596/24599

[13] Abukhader SM Jönson G. Logistics and the environment: is it an established subject? Int J Logist Res Applic. (2004) 7:137–49. 10.1080/13675560410001684201

[14] Fahimnia B Sarkis J Davarzani H. Green supply chain management: a review and bibliometric analysis. Int J Prod Econ. (2015) 162:101–14. 10.1016/j.ijpe.2015.01.003

[15] Abbasi M Nilsson F. Developing environmentally sustainable logistics: exploring themes and challenges from a logistics service providers' perspective. Transport Res Part D Transport Environ. (2016) 46:273–83. 10.1016/j.trd.2016.04.004

[16] Lee SM Choi D Supply chain governance mechanisms, green supply chain management, organizational performance. Sustainability. (2021) 13:13146. 10.3390/su132313146

[17] Oberhofer P Dieplinger M. Sustainability in the transport and logistics sector: lacking environmental measures. Business Strategy Environ. (2014) 23:236–53. 10.1002/bse.176925855820

[18] Dar AA Chen J Shad A Pan X Yao J Bin-Jumah M. A combined experimental and computational study on the oxidative degradation of bromophenols by Fe (VI) and the formation of self-coupling products. Environ Pollut. (2020) 258:113678. 10.1016/j.envpol.2019.113678 31796318

[19] Dar AA Pan B Qin J Zhu Q Lichtfouse E Usman M. Sustainable ferrate oxidation: reaction chemistry, mechanisms and removal of pollutants in wastewater. Environ Pollut. (2021) 290:117957. 10.1016/j.envpol.2021.117957 34425373

[20] Sun Q. Empirical research on coordination evaluation and sustainable development mechanism of regional logistics and new-type urbanization: a panel data analysis from 2000 to 2015 for Liaoning Province in China. Environ Sci Pollut Res. (2017) 24:14163–75. 10.1007/s11356-017-8980-y 28421518

[21] Tang CF Abosedra S. Logistics performance, exports, and growth: evidence from Asian economies. Res Transport Econ. (2019) 78:100743. 10.1016/j.retrec.2019.100743

[22] Ciccone A Hall R. Productivity and the density of economic activity. Am Econ Rev. (1996) 86:54–70.

[23] Hong J. Transport and the location of foreign logistics firms: the Chinese experience. Transport Res Part A Policy Pract. (2007) 41:597–609. 10.1016/j.tra.2006.11.004

[24] Madden G Savage SJ. Telecommunications and economic growth. Int J Soc Econ. (2000) 27:893–906. 10.1108/03068290010336397

[25] Shirley C Winston C. Firm inventory behavior and the returns from highway infrastructure investments. J Urban Econ. (2004) 55:398–415. 10.1016/j.jue.2003.11.001

[26] Roller L-H Waverman L. Telecommunications infrastructure and economic development: a simultaneous approach. Am Econ Rev. (2001) 91:909–23. 10.1257/aer.91.4.909

[27] Civelek ME Uca N Çemberci M. The mediator effect of logistics performance index on the relation between global competitiveness index and gross domestic product. Eur Sci J. (2015) 11:368–75.

[28] Bansal S Sharma GD Rahman MM Yadav A Garg I. Nexus between environmental, social and economic development in South Asia: evidence from econometric models. Heliyon. (2021) 7:e05965. 10.1016/j.heliyon.2021.e05965 33490698 PMC7810780

[29] Murphy PR Poist RF. Green logistics strategies: an analysis of usage patterns. Transport J. (2000) 40:5–16. https://www.jstor.org/stable/20713450

[30] Karaduman HA Karaman-Akgül A Çaglar M Akbaş EH. The relationship between logistics performance and carbon emissions: an empirical investigation on Balkan countries. Int J Climate Change Strat Manag. (2020) 12:449–61. 10.1108/IJCCSM-05-2020-0041

[31] Porter M Van der Linde C. Green and competitive: ending the stalemate. Dyn Eco Effic Econ. (1995) 33.

[32] Khan SAR Qianli D SongBo W Zaman K Zhang Y. Environmental logistics performance indicators affecting per capita income and sectoral growth: evidence from a panel of selected global ranked logistics countries. Environ Sci Pollut Res. (2017) 24:1518–31. 10.1007/s11356-016-7916-2 27785719

[33] Meyer I Leimbach M Jaeger CC. International passenger transport and climate change: a sector analysis in car demand and associated CO2 emissions from 2000 to 2050. Energy Policy. (2007) 35:6332–45. 10.1016/j.enpol.2007.07.025

[34] Khan SAR Zhang Y Anees M Golpîra H Lahmar A Qian li D. Green supply chain management, economic growth and environment: a GMM based evidence. J Clean Prod. (2018) 185:588–99. 10.1016/j.jclepro.2018.02.226

[35] Dong Z Tan Y Wang L Zheng J Hu S. Green supply chain management and clean technology innovation: an empirical analysis of multinational enterprises in China. J Clean Prod. (2021) 310:127377. 10.1016/j.jclepro.2021.127377

[36] Magazzino C Alola AA Schneider N. The trilemma of innovation, logistics performance, and environmental quality in 25 topmost logistics countries: a quantile regression evidence. J Clean Prod. (2021) 322:129050. 10.1016/j.jclepro.2021.129050 PMC9759200 36567950

[37] Tao R Umar M Naseer A Razi U. The dynamic effect of eco-innovation and environmental taxes on carbon neutrality target in emerging seven (E7) economies. J Environ Manag. (2021). 299:113525. 10.1016/j.jenvman.2021.113525 34438310

[38] Sharma GD Shah MI Shahzad U Jain M Chopra R. Exploring the nexus between agriculture and greenhouse gas emissions in BIMSTEC region: the role of renewable energy and human capital as moderators. J Environ Manag. (2021) 297:113316. 10.1016/j.jenvman.2021.113316 34293673

[39] Bhattacharya M Paramati SR Ozturk I Bhattacharya S. The effect of renewable energy consumption on economic growth: evidence from top 38 countries. Appl Energy. (2016) 162:733–41. 10.1016/j.apenergy.2015.10.104

[40] Ehrlich PR Holdren JP. Impact of population growth. Science. (1971) 171:1212–7. 10.1126/science.171.3977.1212 5545198

[41] Paramati SR Alam MS Chen C-F. The effects of tourism on economic growth and CO2 emissions: a comparison between developed and developing economies. J Travel Res. (2017) 56:712–24. 10.1177/0047287516667848

[42] York R Rosa EA Dietz T. Bridging environmental science with environmental policy: plasticity of population, affluence, and technology. Soc Sci Quart. (2002) 83:18–34. 10.1111/1540-6237.00068

[43] Dietz T Rosa EA. Effects of population and affluence on CO2 emissions. Proc Natl Acad Sci USA. (1997) 94:175–9. 10.1073/pnas.94.1.175 8990181 PMC19273

[44] Salim R Yao Y Chen GS. Does human capital matter for energy consumption in China? Energy Econ. (2017) 67:49–59. 10.1016/j.eneco.2017.05.016

[45] Westerlund J. Testing for error correction in panel data. Oxford Bull Econ Stat. (2007) 69:709–48. 10.1111/j.1468-0084.2007.00477.x

[46] Breitung J Pesaran MH. Unit Roots and Cointegration in Panels, in the Econometrics of Panel Data. Springer (2008). p. 279–322. 10.1007/978-3-540-75892-1_9

[47] Moon HR Perron B. Beyond panel unit root tests: using multiple testing to determine the nonstationarity properties of individual series in a panel. J Econ. (2012) 169:29–33. 10.1016/j.jeconom.2012.01.008

[48] Phillips PC Moon HR. Linear regression limit theory for nonstationary panel data. Econometrica. (1999) 67:1057–111. 10.1111/1468-0262.00070

[49] Pesaran MH. A simple panel unit root test in the presence of cross-section dependence. J Appl Econ. (2007) 22:265–312. 10.1002/jae.95125855820

[50] Pesaran MH Yamagata T. Testing slope homogeneity in large panels. J Econ. (2008) 142:50–93. 10.1016/j.jeconom.2007.05.010

[51] Westerlund J Edgerton DL. A simple test for cointegration in dependent panels with structural breaks. Oxford Bull Econ Stat. (2008) 70:665–704. 10.1111/j.1468-0084.2008.00513.x

[52] Chudik A Pesaran MH. Common correlated effects estimation of heterogeneous dynamic panel data models with weakly exogenous regressors. J Econ. (2015) 188:393–420. 10.1016/j.jeconom.2015.03.007

[53] Liddle B. Consumption-based accounting and the trade-carbon emissions nexus in Asia: a heterogeneous, common factor panel analysis. Sustainability. (2018) 10:3627. 10.3390/su10103627

[54] Pesaran MH. Testing weak cross-sectional dependence in large panels. Econ Rev. (2015) 34:1089–117. 10.1080/07474938.2014.956623

[55] Bai J Carrion-I-Silvestre JL. Structural changes, common stochastic trends, and unit roots in panel data. Rev Econ Stud. (2009) 76:471–501. 10.1111/j.1467-937X.2008.00530.x

[56] Su C-W Umar M Khan Z. Does fiscal decentralization and eco-innovation promote renewable energy consumption? Analyzing the role of political risk. Sci Total Environ. (2021) 751:142220. 10.1016/j.scitotenv.2020.142220 33181994

[57] Banerjee A Carrion-i-Silvestre JL. Testing for panel cointegration using common correlated effects estimators. J Time Ser Anal. (2017) 38:610–36. 10.1111/jtsa.12234

[58] Prajogo D Chowdhury M Yeung ACL Cheng TCE. The relationship between supplier management and firm's operational performance: a multi-dimensional perspective. Int J Prod Econ. (2012) 136:123–30. 10.1016/j.ijpe.2011.09.022

[59] Li X Sohail S Majeed MT Ahmad W. Green logistics, economic growth, and environmental quality: evidence from one belt and road initiative economies. Environ Sci Pollut Res. (2021) 28:30664–74. 10.1007/s11356-021-12839-4 33590395

